# RARGE II: An Integrated Phenotype Database of Arabidopsis Mutant Traits Using a Controlled Vocabulary

**DOI:** 10.1093/pcp/pct165

**Published:** 2013-12-20

**Authors:** Kenji Akiyama, Atsushi Kurotani, Kei Iida, Takashi Kuromori, Kazuo Shinozaki, Tetsuya Sakurai

**Affiliations:** ^1^RIKEN Center for Sustainable Resource Science, Yokohama, Kanagawa, 230-0045 Japan; ^2^Graduate School of Medicine, Kyoto University, Kyoto, Kyoto, 606-8501 Japan

**Keywords:** *Arabidopsis*, Database, Gene function, Mutant line, Ontology, Phenotype

## Abstract

*Arabidopsis thaliana* is one of the most popular experimental plants. However, only 40% of its genes have at least one experimental Gene Ontology (GO) annotation assigned. Systematic observation of mutant phenotypes is an important technique for elucidating gene functions. Indeed, several large-scale phenotypic analyses have been performed and have generated phenotypic data sets from many Arabidopsis mutant lines and overexpressing lines, which are freely available online. Since each Arabidopsis mutant line database uses individual phenotype expression, the differences in the structured term sets used by each database make it difficult to compare data sets and make it impossible to search across databases. Therefore, we obtained publicly available information for a total of 66,209 Arabidopsis mutant lines, including loss-of-function (RATM and TARAPPER) and gain-of-function (AtFOX and OsFOX) lines, and integrated the phenotype data by mapping the descriptions onto Plant Ontology (PO) and Phenotypic Quality Ontology (PATO) terms. This approach made it possible to manage the four different phenotype databases as one large data set. Here, we report a publicly accessible web-based database, the RIKEN Arabidopsis Genome Encyclopedia II (RARGE II; http://rarge-v2.psc.riken.jp/), in which all of the data described in this study are included. Using the database, we demonstrated consistency (in terms of protein function) with a previous study and identified the presumed function of an unknown gene. We provide examples of AT1G21600, which is a subunit in the plastid-encoded RNA polymerase complex, and AT5G56980, which is related to the jasmonic acid signaling pathway.

## Introduction

*Arabidopsis thaliana* is one of the most commonly used experimental plants, and many techniques, tools and detailed genomic data are available for working with it. Many studies have been performed on this species. The Arabidopsis Information Resource (TAIR; http://www.arabidopsis.org/) provides a compilation of such information and has enhanced gene annotations by literature curation (Lamesch et al. 2012). TAIR provides controlled vocabulary annotations for Arabidopsis genes, with Gene Ontology (GO) and Plant Ontology (PO) annotations (Cooper et al. 2013) including both experimental and non-experimental evidence (Li et al. 2012). However, despite the efforts of a large number of researchers, only 40% of genes have been assigned at least one experimental GO annotation (Li et al. 2012). To enhance gene function studies, new methods are required to facilitate our understanding of the plant genome.

One direct method for investigating gene function is to examine and characterize the phenotypic changes associated with loss-of-function gene mutations. Insertional mutagenesis with the *Activator* (*Ac*)/*Dissociation* (*Ds*) transposon system makes it possible to generate mutants with a high proportion of single-copy transposon insertions. This system requires the production of a large number of mutant lines to obtain genome-wide coverage. Nonetheless, the single insertion site in each line can be easily determined, thereby simplifying the production and subsequent genetic analysis of single-gene knockout series (Fedoroff and Smith 1993, Sundaresan et al. 1995, Martienssen 1998). On the other hand, gain-of-function mutational analyses provide a separate set of tools that can be used to dissect the functions of genes, especially those with functional redundancy, as found in gene families. Thus, gain-of-function mutants may represent a different spectrum of mutants that have not been isolated as conventional loss-of-function mutants (Nakazawa et al. 2003). In contrast to loss-of-function mutants that cause a recessive phenotype, gain-of-function mutants behave in a dominant manner in the T_1_ generation (Weigel et al. 2000, Bouché and Bouchez 2001). One example of large-scale phenotypic analysis of plant lines generated for gain-of-function mutant screening is the full-length cDNA overexpressor (FOX) gene-hunting system, which is a novel alternative activation tagging technology that uses full-length cDNAs (fl-cDNAs) (Ichikawa et al. 2006). In Arabidopsis, it is possible to conduct gene-based large-scale phenotype analysis from gain-of-function studies using the FOX hunting system (Ichikawa et al. 2006) and to perform loss-of-function studies by saturation mutagenesis (Parinov and Sundaresan 2000).

Several large-scale phenotypic analyses have recently been undertaken, and phenotypic data sets from many Arabidopsis mutant lines and overexpressing lines have been made freely available online (Kuromori et al. 2009). One of these databases, the RIKEN Arabidopsis Phenome Information Database (RAPID; http://rarge.psc.riken.jp/phenome/), houses a total of 4,000 transposon insertion lines, each of which contains a homozygous *Ds* transposon mutation in a gene or promoter region; these genetic lines have been examined systematically for visible phenotypes at various growth stages of the plant (Kuromori et al. 2006). Another database, the Trapper Database (http://genetrap.cshl.org/) from Cold Spring Harbor Laboratory, contains data from 16,000 lines that each carries a unique insertion of a gene trap (GT) or enhancer trap (ET) transposable *Ds* element that both disrupts gene function and can be used to monitor gene expression (Sundaresan et al. 1995, Martienssen 1998). The Arabidopsis FOX (http://nazunafox.psc.database.riken.jp/) and RiceFOX (http://ricefox.psc.riken.jp/) at RIKEN contain a total of 14,000 and 18,000 plants, respectively, that overexpress introduced fl-cDNAs from Arabidopsis and rice based on the FOX hunting system (Ichikawa et al. 2006, Kondou et al. 2009, Sakurai et al. 2011).

Although the Arabidopsis mutant line databases that include phenotypic information are useful, the use of uniquely structured term sets to describe phenotypes in each database makes comparisons of phenotypes among the databases difficult and performing searches across the databases impossible. Therefore, the integration of such databases with a controlled structured vocabulary (ontology) is an effective way to enrich the gene annotation. Furthermore, mapping phenotypic descriptions onto ontology terms may enable the comparison of phenotypes across different species as well as among genes of a single species (Walls et al. 2012). In fact, two databases doing just that have been constructed and made publicly available: PO (http://www.plantontology.org/), which includes information on plant anatomy and development stage (Cooper et al. 2013), and phenotypic quality ontology (PATO; http://obofoundry.org/wiki/index.php/PATO:Main_Page), which includes phenotypic annotations (Mungall et al. 2010).

Here, we describe the integration of the phenotypes of four Arabidopsis mutant lines (including two loss-of-function and two gain-of-function lines) by mapping descriptions into PO and PATO. We have developed an updated version of the RIKEN Arabidopsis Genome Encyclopedia (RARGE), a database of fl-cDNAs and *Ds* transposon mutant lines (Sakurai et al. 2005), which has been designated RARGE II.

## Results and Discussion

### Data sets

Mutant line data including flanking sequence information, phenotype description in text form and images of the mutant lines were obtained from four different sources: 17,198 RIKEN Arabidopsis *Ds* transposon mutant (RATM) lines (Kuromori et al. 2004, Kuromori et al. 2006), 16,337 Cold Spring Harbor Laboratory Arabidopsis Gene trap mutant (TRAPPER) lines (Springer et al. 1995, Martienssen 1998), 14,069 RIKEN Arabidopsis fl-cDNA overexpressed Arabidopsis (AtFOX) lines (Kondou et al. 2010) and 18,605 RIKEN rice full-length cDNA overexpressed Arabidopsis (OsFOX) lines (Kondou et al. 2009, Sakurai et al. 2011) (Table 1).

**Table 1:** Sources of data for the mutant lines

Source	No. of total lines	URL
RIKEN Arabidopsis *Ds* transposon mutant lines	17,198	http://rarge.psc.riken.jp/
Cold Spring Harbor Laboratory Arabidopsis gene trap mutant lines	16,337	http://genetrap.cshl.org/
RIKEN Arabidopsis full-length cDNA overexpressed Arabidopsis lines	14,069	http://nazunafox.psc.database.riken.jp/
RIKEN rice full-length cDNA overexpressed Arabidopsis lines	18,605	http://ricefox.psc.riken.jp/
Total	66,209	

### Deduction of disrupted and induced genes

**Fig. 1:**
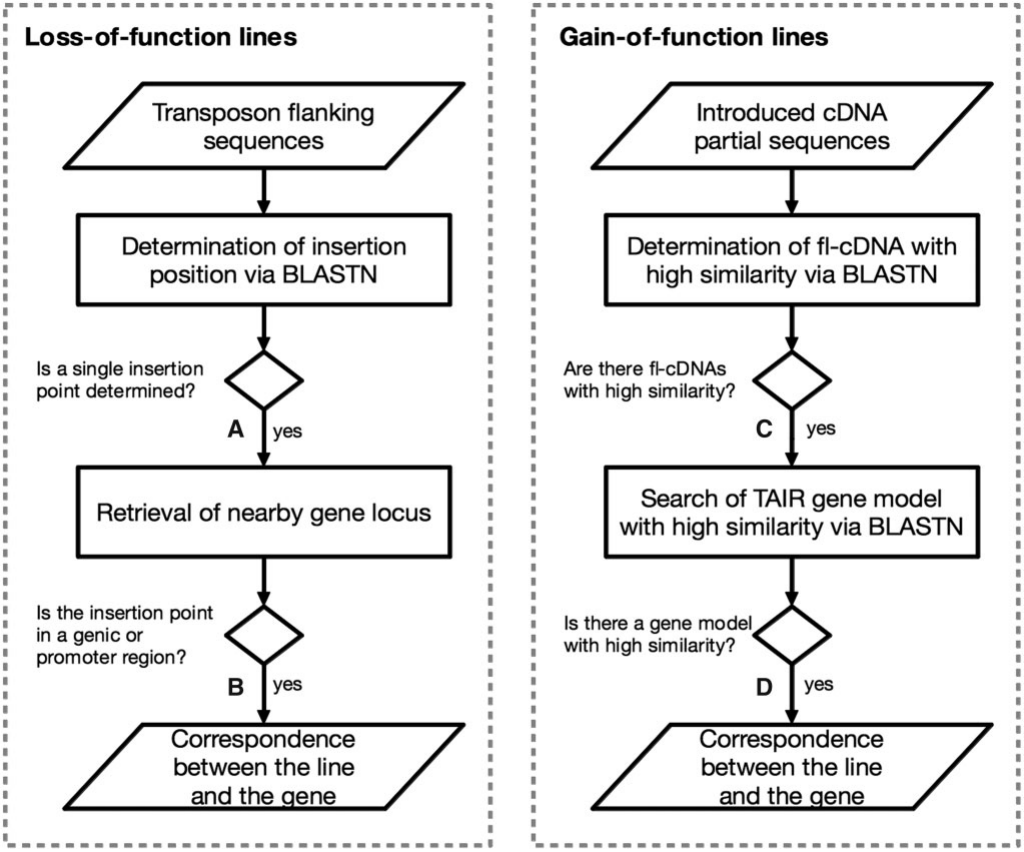
Annotation workflow of loss- and gain-of-function lines. To deduce disrupted and induced genes, we employed independent methods for loss- and gain-of-function lines. For loss-of-function lines (A), we determined a single transposon insertion point and then (B) deduced the genes in which the transposon was inserted into the gene or promoter region. For gain-of-function lines (C), we deduced the introduced full-length cDNAs and then (D) searched for highly similar gene models. Detailed values at each step are described in Tables 1 and 2.

We employed different methods to deduce the genes that were disrupted and induced in the loss-of-function and gain-of-function mutant lines, respectively. Fig. 1 illustrates our workflow to deduce the disrupted and induced genes for each mutant type.

For loss-of-function lines, the single genomic insertion point was determined using a similarity search of transposon-flanking sequences against the TAIR10 Arabidopsis whole-genome sequence (Lamesch et al. 2012) using BLASTN (Altschul et al. 1997) (Fig. 1A); then, gene loci in which the determined insertion point was in the gene or promoter region (Fig. 1B) were retrieved to define disrupted genes. We found that 15,690 (92.8%) of the 17,198 RATM lines and 8,540 (52.3%) of the 16,337 TRAPPER lines had a single insertion point in the genome. Among the loss-of-function lines, 72.2% were determined to have a single transposon insertion point, suggesting that the data set was suitable for this study. Finally, gene loci were retrieved from 13,294 (77.3%) of the RATM lines and 7,184 (44.0%) of the TRAPPER lines (Table 2).

**Table 2:** Disrupted genes in the loss-of-function lines

	RATM	TRAPPER
Total lines	17,198 (139)	16,337 (1,409)
(A) Lines determined to have a single insertion point	15,690 (139)	8,540 (884)
(B) Lines with an insertion in a gene or promoter region	13,294 (139)	7,184 (773)

The number of loss-of-function lines determined to have disrupted genes in each step (see Fig. 1) are shown.

Numbers in parentheses indicate the number of lines observed with any phenotype.

A and B refer to the steps in the workflow shown in Fig. 1.

Introduced fl-cDNAs of gain-of-function lines were determined via a similarity search of partial reading sequences against fl-cDNA sequences using BLASTN (Altschul et al. 1997) (Fig. 1C), and the induced genes were deduced via a similarity search of fl-cDNA sequences against the gene models of TAIR10 (Fig. 1D). OsFOX lines are mutant lines into which rice fl-cDNAs were introduced but, for comparison with the other Arabidopsis mutant lines in this study, a TAIR gene with high similarity was defined as the induced gene. We found that 8,365 (59.5%) of a total of 14,069 AtFOX lines and 11,578 (62.2%) of a total of 18,605 OsFOX lines had introduced fl-cDNAs. Induced genes were deduced in 8,357 (59.4%) of the AtFOX and 10,012 (53.8%) of the OsFOX lines (Table 3). The AtFOX and OsFOX lines contained an average of 2.6 and 1.11 fl-cDNA clones, respectively, per line (Ichikawa et al. 2006, Kondou et al. 2009). In the cases in which multiple fl-cDNAs were detected, we assumed that all fl-cDNAs were overexpressed.

**Table 3:** Overexpressed genes in gain-of-function lines

	AtFOX	OsFOX
Total lines	14,069 (3,429)	18,605 (5,353)
(C) Lines determined to have introduced fl-cDNAs	8,365 (1,670)	11,578 (2,940)
(D) Lines deduced to have induced genes	8,357 (1,668)	10,012 (2,555)

The number of gain-of-function lines determined to have overexpressed genes in each step (see Fig. 1) are shown.

Numbers in parentheses indicate the number of lines observed with any phenotype.

C and D refer to the steps in the workflow shown in Fig. 1.

Next, we calculated the number of the genes corresponding to these lines. The RATM and the TRAPPER lines showed 9,424 (28.3%) and 5,628 (16.9%) disrupted genes, respectively, and the AtFOX and the OsFOX lines showed 2,976 (8.9%) and 4,177 (12.5%) induced genes, respectively. Consequently, data sets were obtained in which loss-of-function lines had 12,558 (37.7%) disrupted genes and gain-of-function lines had 6,382 (19.2%) induced genes. There were 16,015 (48.1%) genes that were disrupted or induced by either loss-of-function or gain-of-function mutations, respectively, and 2,925 (8.8%) genes that were disrupted and induced by both loss-of-function and gain-of-function mutations, respectively (Table 4).

**Table 4:** Summary of the genes disrupted or induced in loss- and/or gain-of-function lines

	Loss-of-function		Gain-of-function	
	RATM	TRAPPER	AtFOX	OsFOX
Genes disrupted or induced per resource	9,424 (159)	5,628 (849)	2,976 (1,176)	4,177 (1,870)
Genes disrupted or induced per mutant type	12,558 (990)		6,382 (2,906)	
Genes disrupted or induced in loss- or gain-of-function lines	16,015 (3,974)			
Genes disrupted and induced in loss- and gain-of-function lines	2,925 (102)			

The numbers of genes disrupted in loss-of-function lines and/or overexpressed in gain-of-function lines are shown.

The numbers in parentheses indicate the number of lines showing any of the observable phenotypes.

### Mapping of phenotype descriptions to ontology terms

All four resources used in this study include data obtained from the observation of plants. To integrate these phenotypic data, we mapped each phenotype description to a pair of PO and PATO terms (Supplementary Tables S1–S4).

Although PO is structured as a multilevel hierarchy, we expressed information on each tissue or developmental stage using up to two PO level terms. As a result, the phenotypic descriptions in the data set were mapped to PO terms organized within 12 classes [*whole plant* (PO:0000003), *seedling* (PO:0008037), *root* (PO:0009005), *vascular leaf* (PO:0009025), *stem* (PO:0009047), *flower* (PO:0009046), *fruit* (PO:0009001), *seed* (PO:0009010), *trichome* (PO:0000282), *0 seed germination stage* (PO:0007057), *whole plant flowering stage* (PO:0007016) and *sporophyte vegetative stage* (PO:0007134)] and 10 subclasses [*cotyledon* (PO:0020030), *hypocotyl* (PO:0020100), *cauline leaf* (PO:0000013), *rosette leaf* (PO:0000014), *stipule* (PO:0020041), *stem internode* (PO:0020142), *petal* (PO:0009032), *sepal* (PO:0009031), *stamen* (PO:0009029) and *gynoecium* (PO:0009062)]. Note that throughout the paper, ontology terms are in italics. PATO terms were organized into 39 classes (Supplementary Table S5). For example, cauline leaf, rosette leaf, and stipule belong to vascular leaf as a higher class.

The numbers of mutant lines observed with phenotypes mapped onto ontology terms are summarized in correspondence with the workflow shown in Fig. 1. Phenotypes were observed for 139 of the RATM lines; all of these were determined as single insertion points of the transposon into the gene or promoter region (Table 2). Phenotypes were observed for 1,409 of the TRAPPER lines, 884 of which were determined to have single insertion points, and the disrupted genes were deduced for 773 of these (Table 2). Phenotypes were observed for 3,429 and 5,353 of the AtFOX and OsFOX lines, respectively, with 1,670 and 2,940 of these lines, respectively, found to contain introduced fl-cDNAs with high similarity. Highly similar Arabidopsis gene models were assigned to 1,668 and 2,555 of the AtFOX and OsFOX lines, respectively (Table 3).

Subsequently, we calculated the number of genes in the same manner as described in the previous section. A total of 159 and 849 of the genes obtained from the RATM and TRAPPER lines, respectively, showed phenotypes by gene disruption; thus, 990 of the genes obtained from the loss-of-function lines had disrupted genes. Similarly, 1,176 and 1,870 genes obtained from the AtFOX and OsFOX lines, respectively, showed phenotypes related to the introduction of fl-cDNAs; thus, 2,906 of the obtained gene gain-of-function lines had these introductions. A total of 3,794 genes showed mutant phenotypes because of either gene disruption or induction; 102 genes showed mutant phenotypes attributable to both gene disruption and induction (Table 4; Supplementary Table S6).

According to Kuromori et al. (2009), many mutants have been assigned as having epinastic leaves in phenotypic data sets of activation-tagged lines, whereas only hyponastic leaf mutants have been registered for transposon-tagged lines (Ichikawa et al. 2003, Kuromori et al. 2006). We found similar results in that many gain-of-function lines were detected using the PO and PATO terms *leaf::epinastic* (PATO:0000945), *cauline leaf::epinastic* and *rosette leaf::epinastic* (note: throughout the paper, pairs of PO and PATO terms are joined with a double colon), whereas very few loss-of-function lines were detected by the terms *rosette leaf::epinastic* (Supplementary Table S5). As another example, several mutants had overgrowth phenotypes (taller plants or high fertility) in the activation-tagged lines, whereas very few such mutants were found in transposon-tagged lines (Ichikawa et al. 2003, Kuromori et al. 2006); several gain-of-function lines were detected by the term *whole plant::increased height* (PATO:0000570), whereas no loss-of-function lines were detected with this term (Supplementary Table S5). Several gain-of-function lines, but no loss-of-function lines, were detected by the term *root::present in greater numbers in organism* (PATO:0000470), whereas several loss-of-function lines, but no gain-of-function lines, were detected by the term *root::present in fewer numbers in organism* (Supplementary Table S5). These examples suggest that some opposite propensities found to date may have depended on the mutation type, such as loss of function and gain of function, rather than the gene category.

### Web interface

**Fig. 2:**
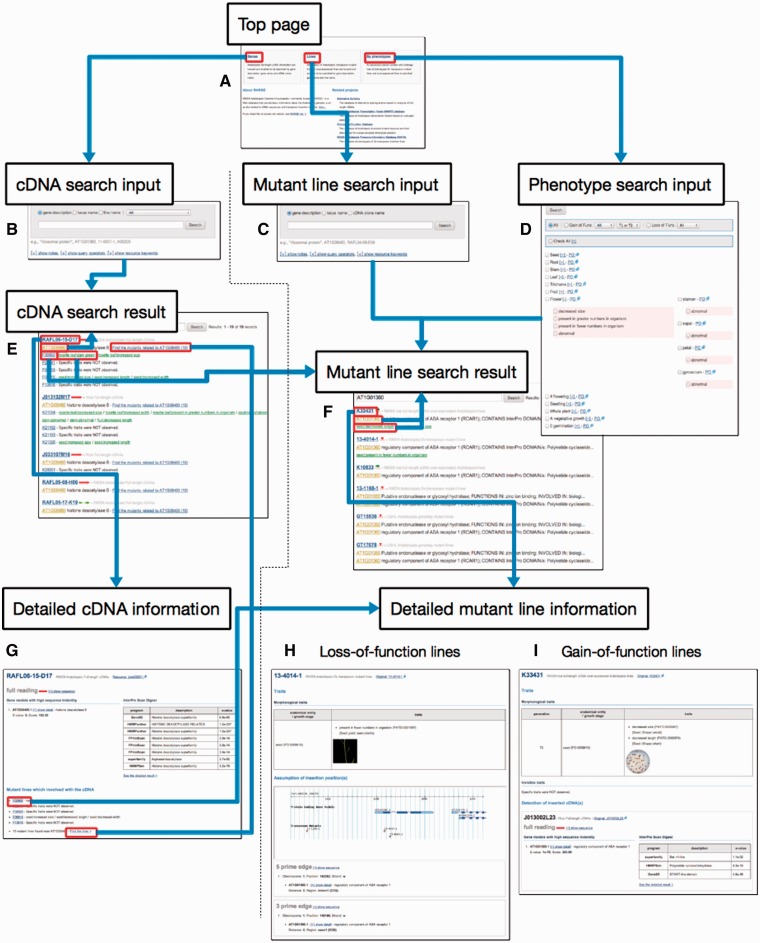
Operation workflow of the web-based database. (A) On the top page, users can select the desired resource, fl-cDNAs or mutant lines, or can find mutant lines by phenotype using the ontology tree. Then they can (B) search fl-cDNAs by keywords with filtering from the fl-cDNA search input page, (C) search for mutant lines by keywords with filtering from the mutant line search input page and/or (D) search all mutant lines by selecting phenotype items in the alternative mutant line search input page by phenotype tree. (E) The fl-cDNA search result page shows records that include the fl-cDNA clone name, gene locus name, gene description, the number of mutant lines with disruption or induction of the gene and the mutant lines into which the fl-cDNA was introduced. (F) The mutant line search result page shows records that include the line name, resource name, deduced disrupted or induced gene locus name, gene description and phenotypes observed. (G) The detailed fl-cDNA information page shows the fl-cDNA sequence, its InterPro Scan result, gene models with high degrees of similarity and the names of mutant lines into which the cDNA was introduced. (H) The detailed mutant line information page for the loss-of-function lines shows the visible phenotypes, including the original phenotype description, mapped PO information and PATO information, line photographs and deduced transposon insertion point. (I) The detailed mutant line information page for the gain-of-function lines shows the visible and invisible phenotypes including the original phenotype description, mapped PO information and PATO information, line photographs, the fl-cDNA sequence, its InterPro Scan result and gene models with high degrees of similarity.

We developed a publicly accessible web-based database, RARGE II (http://rarge-v2.psc.riken.jp/). All data from this study are housed in the database, which provides multifaceted search functions and enables browsing of all mutant lines and phenotype data without restrictions via the Internet using a modern web browser. It was designed to allow seamless, user-friendly viewing of mutant lines and fl-cDNAs (Fig. 2). Users can choose the intended resource, fl-cDNAs or mutant lines, and/or can find mutant lines by phenotype using the ontology tree (Fig. 2A).

Users can search for specific mutant lines by gene description, gene locus name or line name, and can also filter results by selecting the resource name (RATM, TRAPPER, AtFOX and OsFOX) or mutant type (loss-of-function lines and gain-of-function lines) on the mutant search input page (Fig. 2C). The search results page shows the records, including line name, resource name, deduced disrupted or induced gene locus name, gene description and phenotypes observed, making it possible to locate the desired lines easily (Fig. 2F). In addition, gene locus name and phenotypes are linked to the mutant search results queried by the term as the search keyword (Fig. 2F). Each mutant line name in the results is linked to detailed mutant information. A detailed information page shows phenotypes of loss-of-function (Fig. 2H) and gain-of-function lines (Fig. 2I). Phenotype information shown in a table includes the original phenotype description, mapped PO and PATO information and photographs of the mutant line (Fig. 2H, I). With the loss-of-function lines, the flanking sequences, determined insertion point and genome map around the point are shown, allowing the user to deduce the disrupted genes (Fig. 2H). With the gain-of-function lines, the introduced fl-cDNA details are listed on the information page (Fig. 2I).

Users can search all mutant lines by selecting phenotype terms that describe PO and PATO in the tree (Fig. 2D). The terms of only 12 classes of the paired PO are listed and all trees are collapsed. Clicking ‘+’ next to the PO description causes the tree to expand, showing the PO subclasses and PATO (Fig. 2D). Selecting terms by using the checkboxes and performing a search will show search results similar to those of a mutant line search, as described above. In addition, the results can be filtered by selecting a mutant type, a resource name and/or the generation of overexpressed plant (Fig. 2D).

Users can also search fl-cDNAs by gene description, gene locus name and/or fl-cDNA clone name (Fig. 2B). The search results include the records, fl-cDNA clone name, gene locus name, gene description and number of mutant lines with disruption or induction of the gene, especially mutant lines into which an fl-cDNA has been introduced, which are listed with the phenotypes. In addition, the gene locus name and number of mutant lines in which the gene is disrupted or induced are linked to the fl-cDNA search results and mutant search results, respectively, and can be queried by terms such as search keywords (Fig. 2E). Each fl-cDNA clone name in the search results links to detailed fl-cDNA information including the fl-cDNA sequence, its InterPro Scan result, gene models with high similarity, names of mutant lines with the introduced cDNA (that link to the detailed mutant information page) and the number of mutant lines with disrupted or induced gene models that link to the mutant search result page.

The information in our database is widely available to a large number of researchers and will provide the basis for a variety of research projects that rely on large-scale Arabidopsis information. The RARGE II database is easy to use and provides additional important information to the plant biology community, enabling searches for visible phenotypes of genes of interest.

### Comparison of phenotypes among mutant lines with disruption and/or induction of the same gene

Among the loss-of-function lines, we first examined multiple lines in which the mutations were disrupting the same gene. A total of 71 genes had multiple mutant alleles with loss-of-function phenotypes, 45 of which (63%) had at least two alleles showing the same phenotypes (Supplementary Table S7). The most common phenotype was *seed::present in fewer numbers in organism* (93%), followed by *whole plant::decreased height*. On the other hand, when we analyzed multiple mutant lines in which the same gene was induced in gain-in-function lines, 864 genes were obtained, 421 of which (48.7%) showed the same phenotype in more than two independent lines (Supplementary Table S8). Based on past experience, we know that not all individuals in which the same gene was introduced show the same phenotype; in the laboratory, when we investigated the effects of gene transfer, we created many individual overexpressed plants. This study indicated that approximately 50% of genes show gain-of-function phenotypes confirmed by multiple lines; however, the other half seemed to be pseudo-phenotypes.

We wondered what proportion of the mutant lines with related phenotypes among the loss- and gain-of-function lines were affected by the same gene. We compared phenotypes related to 102 of the genes obtained from both loss- and gain-of-function lines that exhibited phenotypes (Supplementary Table S6). In previous phenotype analyses, the fl-cDNAs of desired genes were introduced individually into wild-type plants in both sense and antisense directions. In many instances, the phenotypes of the sense and antisense transgenic lines were discovered to be opposite to those of the gain-of-function and loss-of-function mutants, respectively. In this study, we did not find any exactly opposite phenotypes among loss- and gain-of-function lines except for leaf color. Quantitative traits such as size and length of leaves and roots are caused by multiple factors including the gene expression level. Therefore, these mutant lines had the potential to show other phenotypes even if a disrupted gene was related to a quantitative trait. In other words, it is difficult to define ‘opposite’ and ‘same’ phenotypes. For example, the opposite term for ‘variegated’ cannot be defined as a mutant phenotype because it is likely to be the wild type. The opposite term of ‘large’ is simply ‘small’. However, other phenotypes such as ‘notched’, ‘wrinkled’ and ‘curled’ may appear as opposite phenotypes. The integration of phenotype information in this study does provide a unified phenotype description and a comfortable information environment for deduction of the functions of more unknown proteins. On the other hand, the difficulty in interpreting phenotype descriptions remains.

We sought to determine whether the data set provides new insights for elucidating the functions of genes annotated as ‘unknown protein’. Here, we first present an example that adds supporting information to a previous study on known protein function and then an example of deduction of the function of a gene annotated as an unknown protein.

*Example consistent with a previous study on known protein function.* The AT1G21600 gene is disrupted and induced in the RATM line 15-0303-1 and OsFOX line K06951, which were obtained as a loss-of-function line and a gain-of-function line, respectively (Supplementary Table S6). The protein product, AT1G21600, is a subunit of the plastid-encoded RNA polymerase complex (PTAC6/PAP8), and homozygous seedlings of this line develop white cotyledons, fail to accumulate Chl even under conditions of low light intensity and do not produce primary leaves (Pfalz et al. 2006). The line with a transposon inserted into *AT1G21600*, 15-0303-1, showed an *albino value* phenotype in our data set.

K06951 is an overexpressed line in which the rice fl-cDNA *AK102323* was introduced, which is similar to the Arabidopsis *AT1G21600* gene. To the best of our knowledge, there have been no studies regarding overexpression of the *AT1G21600* gene. Our data for K06951 include several phenotypic descriptions of the cauline leaves, rosette leaves and stem. We found a change in the rosette leaf color to dark green, strongly suggesting a relationship between loss-of-function and gain-of-function phenotypes. Analysis of K06951 would help to elucidate the function of AT1G21600 further.

*Example deducing the function of a gene annotated as an unknown protein.* First, we defined an unknown protein as a protein encoded by a gene that does not have a high degree of similarity to well-known genes and does not have known protein functions, including interaction, localization and enzyme activity. The *AT5G56980* gene was found to encode an unknown protein that is disrupted and induced in the lines GT4800 and F21325, which were obtained as a loss-of-function line and a gain-of-function line, respectively (Supplementary Table S6). GT4800 is a line with a transposon insertion into *AT5G56980* and shows various phenotypes, such as fewer numbers of seeds, decreased plant height and seedling lethality. F21325 is the overexpressed line with introduction of Arabidopsis fl-cDNA *AF385692*, corresponding to *AT5G56980*; it showed an abnormal morphological feature, i.e. rosette leaves.

The *AT5G56980* gene was identified as a pathogen-associated molecular pattern-induced gene (*A70*), which is rapidly induced in systemic tissue after challenge with *Pseudomonas syringae* pv. *tomato* DC3000(*avrRpm1*) but not with the compatible DC3000 strain (Truman et al. 2007). Although *AT5G56980* expression was found to be responsive to jasmonic acid (JA) and wounding, but unaffected by heat, cold or salicylic acid treatment, the function of the protein product of AT5G56980 is unclear (Truman et al. 2007). An example of the JA-related gene, *coi1*, is a principal component of a JA receptor in Arabidopsis and other plants (Xie et al. 1998, Katsir et al. 2008, Yan et al. 2009), and OsCOI1-RNAi (RNA interference) plants show increased plant height and cellular elongation (Yang et al. 2012). If JA is normally controlled via negative feedback by AT5G56980, the GT4800 phenotype *whole plant::decreased height* would be consistently described inversely with OsCOI1-RNAi plants. If the above assumptions are correct, further analyses of GT4800 and F21325 would be worthwhile.

### Perspective

We integrated phenotype information using ontologies from four large-scale mutant databases. As the results of this strategy helped to deduce the function of a gene annotated as an unknown protein, the integration of additional data regarding mutant phenotypes may facilitate deduction of the functions of more unknown proteins. The majority of Arabidopsis insertion mutant lines are transfer DNA-tagged (T-DNA-tagged) lines. However, a systematic phenotype analysis of such lines has not been available to date. Systematic phenotype analysis targeting T-DNA-tagged lines would markedly improve the accuracy of deduction of unknown protein functions. Other types of phenotypic information are also important. For example, the Chloroplast Function Database focuses on nuclear-encoded chloroplast proteins (Myouga et al. 2010, Myouga et al. 2013), and the RiceFOX database houses invisible phenotype information such as photosynthesis activity, plant hormone accumulation and stress sensitivity (Sakurai et al. 2011). The Plant Organelles Database (Mano et al. 2014) contains an electron micrograph database and an organelles database for various plant species and mutant lines. Comprehensive analyses of metabolites and plant hormones (metabolomics and hormonomics) have become a rapidly developing research field in recent years. For example, comprehensive measurement data for metabolites and plant hormones are publicly available in PRIMe (http://prime.psc.riken.jp/) (Akiyama et al. 2008, Sakurai et al. 2013) and UniVIO (http://univio.psc.riken.jp/) (Kudo et al. 2013), respectively. A database of the relationships between species and metabolites has been developed in KNApSAcK (http://kanaya.naist.jp/KNApSAcK_Family/) (Afendi et al. 2012, Nakamura et al. 2013). We expect that invisible phenotypes will be important resources for mutant analysis in the future. It is significant that the information resources stated above are appended to the RARGE II database. On the other hand, an approach to extract phenotypic descriptions manually from the literature has been developed by TAIR (Li et al. 2012). In addition, the achievements of our exhaustive research and many individual studies reported in the literature should be integrated. Furthermore, although we used only visible phenotypes in this study, we will integrate invisible phenotypes such as chemical and biological phenotypes in future studies as more data on these phenotypes become available every year.

## Materials and Methods

### Data sets

The data sets of mutant line information were obtained from the public databases detailed in Table 1. RATM, AtFOX and OsFOX data were obtained directly from each database. TRAPPER data were obtained using Web Crawler. The PO data file (version #19) was obtained from http://palea.cgrb.oregonstate.edu/viewsvn/Poc/tags/live/plant_ontology.obo. The PATO data file [21:05:2013/14:51 (date/time last created)] was obtained from http://pato.googlecode.com/svn/trunk/quality.obo.

### Deduction of disrupted genes in loss-of-function lines

Transposon flanking sequences were subjected to a BLASTN search against the Arabidopsis genome sequence from the TAIR10 genome release (Lamesch et al. 2012). The position showing the highest similarity score with an E-value <1e-15 was defined as an insertion point. When all insertion points of multiple flanking sequences were mapped within 200 bp, it was defined as a single insertion point. When a transposon was inserted into a gene or promoter region, we picked the gene locus name. The 1,000 bp of upstream sequence from each transcriptional start site was defined as the promoter region. When a transposon was inserted into the gene or promoter regions of multiple genes, both genes were selected.

### Deduction of induced genes in gain-of-function lines

The partial sequences of the introduced fl-cDNAs were subjected to a BLASTN search against Arabidopsis fl-cDNA sequences (Seki et al. 2002) or rice fl-cDNA sequences (Kikuchi et al. 2003). The fl-cDNA showing the highest similarity score was defined as an introduced fl-cDNA. The sequences of introduced fl-cDNAs were subsequently subjected to a BLAST search against the Arabidopsis gene model sequences from the TAIR10 genome release (Lamesch et al. 2012). We picked the gene model name showing the highest degree of similarity and an E-value <1e-5.

### Mapping of phenotype description to ontology

We manually mapped the original phenotype descriptions to ontology terms paired with PO (Cooper et al. 2013) and PATO (Gkoutos et al. 2005) based on recorded observations about each line as well as photographs and the literature. For example, the phenotype descriptions ‘Seed yield::low’, ‘Seed yield::semi sterile’ and ‘Seed yield::sterile’ in AtFOX; ‘Seed::Number::few’ in OsFOX; ‘Seed yield::low yield’, ‘Seed yield::semi-sterile’ and ‘Seed yield::sterile’ in RATM; and ‘conditional male sterile’, ‘embryo lethal’, ‘semi-sterile’, ‘sterile’ and ‘very few seeds’ in TRAPPER were mapped to the PO term *seed* (PO:0009010) and the PATO term *present in fewer numbers in organism* (PATO:0001997).

### Database construction

The integrated phenotypic data were stored in the database and implemented in the web application. All programs for manipulating data, such as those used to query the database and generate web pages, were written in Perl (http://www.perl.org/) with the web application framework Catalyst (http://www.catalystframework.org/) and the web server interface PSGI/Plack (http://plackperl.org/). The relational database management system was MySQL (http://www.mysql.com/). This web application system is capable of running on a UNIX-like operating system such as Linux (http://www.linux.org/). The web pages were written in HTML, cascading style sheet and Javascript with the YUI library (http://yuilibrary.com/).

## Supplementary data

Supplementary data are available at PCP online.

## Funding

This work was supported by the Japan Society for the Promotion of Science [a Grant-in-Aid for Young Scientists (B) (18700106 to T.S.)].

## Supplementary Material

Supplementary Data

## Abbreviations

*Ac*/*Ds*: *Activator*/*Dissociation*
AtFOX: RIKEN Arabidopsis full-length cDNA overexpressed Arabidopsis lines
fl-cDNA: full-length cDNA
FOX: full-length cDNA overexpressor
GO: Gene Ontology
JA: jasmonic acid
OsFOX: RIKEN rice full-length cDNA overexpressed Arabidopsis lines
PATO: Phenotype Quality Ontology
PO: Plant Ontology
RARGE: RIKEN Arabidopsis genome encyclopedia
RATM: RIKEN Arabidopsis *Ds* transposon mutant lines
RNAi: RNA interference
TAIR: the Arabidopsis information resource
TRAPPER: Cold Spring Harbor Laboratory Arabidopsis gene trap mutant lines
