# Supplementary material for: RARGE II: An Integrated Phenotype Database of Arabidopsis Mutant Traits Using a Controlled Vocabulary
Source: Plant Cell Physiol. 2013 Dec 20;55(1):e4. doi: 10.1093/pcp/pct165 (PMC3894705; doi:10.1093/pcp/pct165)
Supplement: Supplementary Data [file supp_55_1_e4__index.html]

RARGE II: An integrated phenotype database of Arabidopsis mutant traits using a controlled vocabulary — RARGE II: An Integrated Phenotype Database of Arabidopsis Mutant Traits Using a Controlled Vocabulary — RARGE II: An Integrated Phenotype Database of Arabidopsis Mutant Traits Using a Controlled Vocabulary — Supplementary Data 

# RARGE II: An Integrated Phenotype Database of Arabidopsis Mutant Traits Using a Controlled Vocabulary

## Supplementary Data

files

**Files in this Data Supplement:**

- Supplementary Data - xls file
- Supplementary Data - xls file
- Supplementary Data - xls file
- Supplementary Data - xls file
- Supplementary Data - xls file
- Supplementary Data - xls file
- Supplementary Data - xls file
- Supplementary Data - xls file
